# The Role of Apoptotic Factors in Assessing Progression of Periodontal Disease

**DOI:** 10.19070/2377-8075-1600064

**Published:** 2016-09-03

**Authors:** D Dabiri, S Halubai, M Layher, C Klausner, H Makhoul, GH Lin, G Eckert, H Abuhussein, P Kamarajan, Y Kapila

**Affiliations:** 1 Department of Periodontics and Oral Medicine, School of Dentistry, University of Michigan, USA; 2 Department of Surgical Sciences, Marquette University School of Dentistry, USA; 3 Department of Biostatistics, Indiana University, USA; 4 Department of Orofacial Sciences, School of Dentistry, University of California San Francisco, USA

**Keywords:** Apoptosis, Periodontal Disease, Caspase-3, Fas, FasL, Gingival Crevicular Fluid

## Abstract

**Background:**

The mechanisms responsible for periodontal disease progression remain unclear. However, recent studies suggest that apoptosis may be one mechanism underlying the pathophysiology of periodontal disease progression. This pilot study is the 3 month follow-up of our published baseline study on the presence of apoptotic factors in serum, saliva, and gingival crevicular fluid (GCF) and their association with periodontal disease severity and activity.

**Methods:**

GCF samples were obtained from 37 adult patients with chronic periodontitis (CP) and 7 healthy controls. Clinical measurements, including probing depth (PD), clinical attachment level (CAL), and radiographs, were used to evaluate data by sites and to classify patients into healthy, mild, and moderate/severe CP groups. Enzyme-linked immunosorbent assays were used to measure apoptosis or DNA fragmentation levels in GCF. Western immunoblotting was used to detect several apoptotic proteins, Fas, FasL, sFasL, and caspase-3 expression and its cleavage products in GCF.

**Results:**

At the patient level clinical and apoptotic measurements change minimally over time. At the site level, DNA fragmentation levels increase with increasing PDs at 3 months and baseline. Apoptotic protein expression exhibits increasing trends with increasing PDs at baseline and 3 months. FasL and Active FasL show a high specificity and PPV; low sensitivity and NPV. Caspase-3 products (ProCas35K and Active Cas) show a high PPV with moderate to high specificity; low sensitivity and NPV. ProCas70K shows a high PPV with moderate to high sensitivity; low specificity and NPV.

**Conclusion:**

Factors associated with apoptosis show minimal changes in expression in periodontitis groups in comparison to a healthy group over a short time interval (3 months). However, at the site level, apoptotic factors (DNA fragmentation and apoptotic proteins) exhibit significant increases or increasing trends with increasing PDs at any time point examined (baseline or 3 months). Several of these apoptotic factors also exhibit a high sensitivity and high positive predictive value. Thus, apoptotic molecules may be helpful biomarkers of disease status at any point in time.

## Introduction

The mechanisms responsible for periodontal disease progression remain unclear. However, recent studies suggest that apoptosis or programmed cell death may be one mechanism underlying the pathophysiology of periodontal disease progression [1, 2]. Apoptosis plays a critical role in the regulation of the host immune response and inflammation and can be modulated by various stimuli, including cytokines, bacterial and viral infections, immune cells themselves, and changes in growth factors, nutrients, and the extracellular matrix [3].

One of the best-defined apoptotic pathways is mediated by the death receptor Fas and Fas ligand (FasL) complex. The Fas receptor is expressed on many cell types that include gingival fibroblasts, skin keratinocytes, and T cells [4–6]. Apoptotic cells are present in diseased gingiva [7]. During cell apoptosis, a sequential activation of cysteine proteases, called caspases, plays a central role in the execution phase of apoptosis. Caspase-3 is one of the key executor caspases that regulates a number of critical cellular substrates and when active has been easily detected in cells undergoing apoptosis [8]. Eventually, the caspase cascade culminates in the cleavage of DNA into fragments, a hallmark of apoptosis.

Previously, we reported that factors associated with apoptosis were detected in GCF in patients with chronic periodontitis [9]. Specifically, DNA fragmentation was positively correlated with probing depth (PD) and clinical attachment loss (CAL) regardless of patient disease status (P<0.001). sFas and sFasL were present in saliva and serum, but there were no differences between groups. In GCF, the greater odds of detecting Fas, sFasL, and caspase-3 increased with increasing PD and CAL (P<0.05). In addition, sites with inflammation and PD ≥5 mm had significantly greater odds of exhibiting Fas, sFasL, and caspase-3 expression compared with sites without inflammation and PD<5 mm (P <0.05). Caspase-3 was not detected in saliva or serum. At the patient level, only FasL and disease status were significantly correlated (P<0.05).

The purpose of the current study is to extend these findings and further define the role of these apoptotic factors in assessing the progression of periodontal disease.

## Material and Methods

### Study Design

A single-center longitudinal study of patients with or without periodontal disease was conducted at the Michigan Center for Oral Health Research at the University of Michigan from 2006 to 2009 after University of Michigan Institutional Review Board approval was obtained. Subject data was collected at baseline, 3 months and 6 months. Subject data collected included clinical measurements, GCF, saliva, serum, and standardized dental radiographs. All subject data were collected at baseline, 3 months, and 6 months, except for dental radiographs, which were collected at baseline and 6 months. Non-surgical periodontal therapy consisting of a periodontal prophylaxis or scaling and root planning was completed for each subject at the conclusion of the study at 6 months. Rescue therapy was provided for any periodontal site that exhibited increases in clinical attachment loss greater than or equal to 3 mm. Baseline data results and collection methods for the biofulids (GCF, saliva, and serum) were previously reported [9]. In the current report, longitudinal comparisons were made between the baseline and 3 month data, specifically for the clinical measurements and GCF.

Fifty-seven adult patients (26 males and 31 females, aged 28 to 79 years; mean age: 52.45 years) were recruited from the surrounding community after giving written consent (Table 1). Participants were excluded from the study if they: 1) had < 20 teeth; 2) were pregnant or suffered from metabolic bone diseases, auto-immune disease, uncontrolled diabetes, or post-menopausal osteoporosis; 3) had a history of periodontal therapy; or 4) received antibiotics or long-term non-steroidal anti-inflammatory drugs within the past 3 months. Participants were assigned to one of three groups according to criteria described by Ramseier et al., [10] 1) a healthy/gingivitis group with ≤3-mm clinical attachment level/ loss (CAL), no probing depth (PD) ≥4 mm, and no radio-graphic alveolar bone loss; 2) a mild CP group with at least four sites with radiographic bone loss, PD ≥4 mm, and < 30% of sites with CAL > 3 mm; and 3) a moderate/severe CP group with at least four sites with radiographic bone loss, PD ≥4 mm, and > 30% of sites with CAL > 3 mm.

### Clinical Measurements

Inter-examiner calibration was performed among examiners (HA, ML, and CK) on a periodic basis, and a k score of 0.8 or more was maintained throughout the study. All teeth except third molars were assessed for periodontal clinical measures. Clinical parameters, including PD, CAL, and bleeding on probing (BOP), were measured at six sites per tooth. Other clinical measures, included dichotomous measures of plaque accumulation index (PI) and gingival redness index (GI) as described previously by Haffajee et al., [11].

### GCF Collection and Sampling

GCF from the mesio-buccal site of each tooth was collected using paper strips. To minimize contamination, all clinically detectable supragingival plaque was removed, and the area was isolated with cotton rolls and gently air dried before sampling. During sampling, the paper strip was inserted below the gingival margin until slight resistance was felt, held in place for 30 seconds, and then removed.

### GCF volume was measured using a calibrated machine (Periotron, Oraflow Inc)

Strips that were contaminated with saliva, blood, or plaque were discarded. After volume determination, strips were stored in sterile collection tubes that were labeled with tooth numbers and contained a pre-aliquoted protease inhibitor cocktail and then stored at −8°C. Before analysis, the absorbed fluid was eluted from each strip into 135 mL lysis buffer (100 mL 1 mM phenylmethylsulfonyl fluoride in 1 mL radioimmunoprecipitation assay buffer).

### Sample Analyses

#### Enzyme-linked immunosorbent assays

For DNA fragmentation detection, the eluted GCF samples were assayed for histone-associated DNA fragments using an ELISA detection kit according to manufacturer's instructions. (R&D Systems, Minneapolis, MN.)

#### Western immunoblotting

Western immunoblotting was used to detect the expression of specific apoptotic proteins in the GCF samples. Briefly, 50-mL of eluted GCF sample was mixed with 50 mL sample buffer (950 mL Laemmli sample buffer plus 50 mL 2-mercaptoethanol), boiled for 5 minutes, and divided into three aliquots of equal volume. The molecular weight markers, controls (human oral squamous cell carcinoma cells treated with cisplatin), and equal volumes of samples (one tooth per well) were loaded onto 10% to 12% sodium dodecyl sulfate polyacrylamide gels and electrophoretically resolved under standard conditions.

Next, the proteins were transferred onto polyvinylidene difluoride membranes using standard protocols. After transfer, the membranes were incubated for 45 minutes in Blotto-TBS, Thermo Fisher Scientific Tris-buffered saline (TBS) to block non-specific protein binding sites. Membranes were washed three times for 10 minutes with TBS and polysorbate 20 (TBST), separately incubated with 1:400 dilution of mouse antihuman Fas, mouse anti-human FasL, and mouse antihuman caspase-3 overnight at 4°C, and then washed in TBST three times for 10 minutes each. Membranes were incubated with horseradish peroxidase-labeled anti-mouse immunoglobulin G for 2 hours at room temperature, and chemiluminescent peroxidase substrate was applied according to the instructions of the manufacturer. Membranes were briefly exposed to radiographic film, and densities of bands were scanned and analyzed using a scanning densitometer.

Variations in the volume and protein sample loaded were corrected by assessing the targeted apoptotic proteins using a binary scale. The presence of each protein band was scored and classifiedas negative or positive based on comparison with positive controls in accordance with the technique used by Bersano et al. The presence of each protein (Fas, FasL, and active caspase-3) was counted if a band at a specific molecular weight per antibody was detected and was consistent with the positive control [12].

### Data Analyses

The sample size calculation of 50 participants was based on the original baseline data. Clinical measurements, including PD, CAL, and radiographs, were used to classify patients into healthy, mild, and moderate/severe CP groups.

Data analysis was performed using the Statistical Package for the Social Sciences (SPSS) software. The unit of analysis was tooth site, but the fact that sites were grouped within a patient was also accounted for. The level of significance was set at P <0.05. All data were expressed as mean ± SE for each factor.

Linear mixed models were used to compare the baseline and 3-month PD and CAL data. The models included fixed effects for time, patient type, and the time-by-type interaction. Random effects were included to account for the correlations between the two times and correlations among teeth. Similar generalized linear mixed models were used for comparisons of all other variables, which were evaluated for presence or absence at the two times.

## Results

### Demographic Distribution of Patients at the 3-month recall

Thirty-seven patients returned for their 3-month recall visit: 20 females and 17 males. There were 7 in the healthy/gingivitis group, 19 in the mild CP group, and 11 in the moderate/severe CP group (Table 1). There were 13 smokers versus 24 non-smokers. Ethnicity distributions were as follow: 28 Caucasian, 4 Asian, 1 African-American, 1 Hispanic and 3 others. Regression analysis was performed to account for possible confounding effects, although this did not appear to influence the results. PD, CAL, PI, BOP, and GI in the healthy group were significantly less than in the periodontitis groups (P < 0.001).

### Clinical and Apoptotic Measurements by Patient Group Change Minimally over time

As illustrated in Tables 2 and 3, PD increased significantly from baseline to 3 months for mild CP (p=0.004) and decreased significantly from baseline to 3 months for moderate/severe CP (p<0.001), but did not change significantly for healthy (p=0.07). CAL increased significantly from baseline to 3 months (p=0.045), but the amount of change did not differ significantly by patient type (p=0.40). PI decreased significantly from baseline to 3 months for healthy (p=0.016) but did not change significantly for mild CP (p=0.07) or moderate/severe CP (p=0.51). FasL increased significantly from baseline to 3 months for moderate/ severe CP (p<0.001) but did not change significantly for healthy (p=0.60) or mild CP (p=0.06). Active FasL decreased significantly from baseline to 3 months for healthy (p<0.001) but did not change significantly for mild CP (p=0.37) or moderate/severe CP (p=0.49). ProCas 35K decreased significantly from baseline to 3 months (p<0.001), but the amount of change did not differ significantly by patient type (p=0.52). Active Cas decreased significantly from baseline to 3 months (p < 0.001), with a larger decrease for healthy and moderate/severe CP than for mild CP (p=0.002). DNA fragmentation/ELISA values decreased significantly from baseline to 3 months for mild CP (p<0.001) and moderate/severe CP (p<0.001) but did not change significantly for healthy (p=0.57). BOP (p=0.52), GI (p=0.49), and ProCas70K (p=0.60) did not change significantly from baseline to 3 months.

### DNA Fragmentation Levels Increase with Increasing PDs at Baseline and 3 months

Tables of the mean DNA fragmentation values by PD for Baseline and 3 months are represented (Tables 4 and 5). Comparisons between PDs were made using ANOVA with a fixed effect for PD and a random subject effect to account for correlations among sites within a subject. A Tukey multiple comparisons adjustment was used for the pairwise comparisons between PDs. There were statistically significant increases in DNA fragmentation in the larger PDs at Baseline, including PDs of 1 versus 4 (p=0.0011), 1 versus 5 (p=0.0089), 1 versus ≥6 (p<0.0001), 2 versus 4 (p<0.0001), 2 versus 5 (p=0.0008), 2 versus ≥6 (p<0.0001), 3 versus 4 (p=0.0214), 3 versus ≥6 (p=0.0004). Similarly, at 3 months, DNA fragmentation was significantly higher for PDs≥6 in comparison to the shallower PDs. In addition, shallower PDs showed a trend toward increasing DNA fragmentation with increasing PDs.

### Apoptotic Protein Expression Values Trend to Increase with increasing PDs at Baseline and 3 months

Tables of the FasL, Active FasL, ProCas 35K, ProCas 70K, and Active Cas percentages by PD for Baseline and 3 months are represented (Tables 6 and 7). Comparisons between PDs were made using generalized estimating equations (GEE) applied to logistic regression, with a fixed effect for PD and a clustering subject effect to account for correlations among sites within a subject. A Tukey multiple comparisons adjustment was used for the pairwise comparisons between PDs. The data showed that at baseline there was no significant differences among groups for FasL (p=0.47), Active FasL (p=0.47), ProCas 35K (p=0.20), ProCas 70K (p=0.27), or Active Cas (p=0.11). However, there was a general trend toward increasing values with increasing PDs especially for ProCas 35K, ProCas 70K, and active Cas. Similary, at 3 months, there was no significant differences among groups for FasL (p=0.23), Active FasL (p=0.13), ProCas 70K (p=0.71), ProCas 35K (except for PD 1 vs 3) or Active Cas (p=0.28). However, there was increased expression of active FasL across time by 20% and an increase in Active Cas for the deepest pockets (PD≥6) across time.

### Correlations between pocket depth and apoptosis are significant but small

Table 8 shows the site level data merged with all of the patient-level data for each site level observation. The main reason to merge in the patient-level data was to ensure that sex, age, race, and smoking status were accounted for in the site-level data. Thus, this correlation table represents correlations using the site-level data for pocket depth versus the DNA fragmentation and apoptosis variables. Although most of the correlations are statistically significant, they are generally small.

### Analyses of sensitivity of GCF Markers at Baseline vs 3 months

Regardless of the time point, FasL and Active FasL had higher specificity and positive predictive values (PPV) in comparison to the other apoptotic protein markers (Table 9). At 3 months FasL had high specificity (85.4%) and PPV (92.8%) with low sensitivity (49.4%) and low negative predictive value NPV (38.1%). In comparison, baseline FasL still had 83.2% specificity and 89.6% PPV. Pro caspase-3 (35 kDa) and Active Caspase-3 had high PPV (88.9% and 87.1% respectively at 3 months) with moderate to high specificity with low sensitivity and NPV. Pro Caspase-3 (70 kDa) had a high PPV with moderate to high sensitivity and a low specificity and NPV.

## Discussion

GCF is an inflammatory exudate that seeps into gingival crevices or periodontal pockets around teeth. Since 1960, there has been great interest in the diagnostic potential of GCF due to its composition; namely serum and locally generated biomolecules, such as tissue breakdown products, inflammatory mediators, and antibodies directed against dental plaque bacteria. Analysis of GCF has been studied as a way to quantitatively evaluate the inflammatory status of gingival and periodontal tissues.

Chronic periodontitis is a multifactorial infection caused by a complex group of bacterial species that release inflammatory mediators by interaction with host tissues and cells; some of these mediators cause destruction of the periodontal tissues [13]. In recent years, there has been growing evidence to support the concept that the pathogenesis of infectious diseases is largely mediated by apoptosis triggered by bacteria [14]. Apoptosis of host cells is directly induced by specific pathogens or their extracellular products. Caspase-3 is one of the key executioners of apoptosis within host cells [15].

Apoptosis has been shown in other periodontal studies in relation to various gene expression patterns. Papapanou PN. et al., showed limited differences between gingival tissue transcriptional profiles of aggressive periodontitis and chronic periodontitis, with genes related to immune responses, apoptosis, and signal transduction over expressed in aggressive periodontitis, and genes related to epithelial integrity and metabolism were overexpressed in chronic periodontitis [16]. Johnson et al., looked at the gender differences in inflammatory and apoptotic signaling molecules in normal and diseased human gingiva by assessing Caspase-3 expressions [17]. According to their study, they found females had the highest incidence of gingival apoptosis at sites of periodontal disease.

Pradeep et al., showed that the GCF and serum concentration of caspase-3 proportionally increases with the progression of periodontal disease, that is, gingival inflammation, PD and CAL[18]. In order to to quantitatively assess the presence of apoptotic cells in patients with periodontal disorders, Janbring et al., measured the by-products of apoptotic pathways by looking at DNA tags (DNA Fragmentation assay) [10]. Their results showed that in the most apical part of the sulcus, close to the junctional epithelium, the number of apoptotic keratinocytes exceeded the proliferative ones in patients with periodontitis.

Therefore, a combination of DNA fragmentation and apoptotic protein biomarkers, such as caspase-3 may be useful in assessing site-specific disease over time. However, when looking at the data at the patient level it appears that the significance and correlation of the biological data to the clinical presentation become diluted by the mass effect of patient variables. Given that the relative overall disease level for this population (a Recall population) was low, comparisons across patient groups was not as beneficial as using a site specific analysis over time. Measurement error can also play an important role in this effect. These errors can be large enough to substantially distort apparent site-specific outcomes. The magnitude and frequency of measurement errors, including angulation of the probe; type and location of the tooth and site; and patient characteristics can all affect site-specific and episodic nature of disease activity coupled with the clinician's measurement error within one to two millimeters in using the periodontal probe [19]. In summary, several of the apoptotic factors exhibit a high sensitivity and high positive predictive value for periodontal disease. Thus, apoptotic molecules may be helpful biomarkers of disease status at any point in time.

## Figures and Tables

**Table 1:** Baseline characteristics of subjects with 3-month apoptosis data.

		All	Healthy	Mild CP	Mod/Sev CP
Gender	Female	20 (54%)	6 (86%)	12 (63%)	2 (18%)
	Male	17 (46%)	1 (14%)	7 (37%)	9 (82%)
Current smoking status	N	24 (65%)	6 (86%)	15 (79%)	3 (27%)
	Y	13 (35%)	1 (14%)	4 (21%)	8 (73%)
Ethnicity	African-American	1 (3%)	0 (0%)	0 (0%)	1 (9%)
	Asian	4 (11%)	0 (0%)	2 (11%)	2 (18%)
	Caucasian	28 (76%)	5 (71%)	15 (79%)	8 (73%)
	Hispanic	1 (3%)	0 (0%)	1 (5%)	0 (0%)
	Other	3 (8%)	2 (29%)	1 (5%)	0 (0%)

	All (n=37)			Healthy (n=7)			Mild CP (n=19)			Mod/Sev CP (n=11)		
	Mean (SD)	Min	Max	Mean (SD)	Min	Max	Mean (SD)	Min	Max	Mean (SD)	Min	Max
Age	51.92 (1.81)	28	79	47.86 (5.22)	28	70	51.53 (2.11)	39	66	55.18 (3.63)	37	79
PD	2.51 (0.11)	1.45	4.02	1.65 (0.06)	1.45	1.91	2.40 (0.08)	1.79	2.97	3.25 (0.14)	2.69	4.02
PD≥4	0.20 (0.03)	0	0.63	0.01 (0.00)	0	0.03	0.16 (0.02)	0	0.3	0.39 (0.04)	0.19	0.63
AL	2.15 (0.19)	0.03	4.98	0.70 (0.23)	0.03	1.36	1.90 (0.11)	0.93	2.83	3.51 (0.21)	2.59	4.98
BOP	0.52 (0.04)	0.10	1	0.21 (0.04)	0.10	0.32	0.55 (0.06)	0.1	1	0.65 (0.05)	0.42	0.83
GI	0.70 (0.04)	0.25	1	0.45 (0.07)	0.25	0.71	0.74 (0.05)	0.29	1	0.80 (0.05)	0.55	1
PI	0.66 (0.04)	0.21	0.99	0.32 (0.05)	0.21	0.58	0.75 (0.04)	0.39	0.99	0.73 (0.06)	0.24	0.98
Num Sites	152.38 (2.26)	120	168	162.00 (2.27)	156	168	151.84 (2.73)	132	168	147.18 (5.30)	120	168
Num Teeth	25.51 (0.37)	20	28	27.00 (0.38)	26	28	25.42 (0.45)	22	28	24.73 (0.91)	20	28

**Table 2:** Change from baseline to 3 months for clinical measurements.

	Time	All		Healthy		Mild CP		Mod/Sev CP	
		N Sites	Mean (SE) or N (%)	N Sites	Mean (SE) or N (%)	N Sites	Mean (SE) or N (%)	N Sites	Mean (SE) or N (%)
PD	BL	5604	2.485 (0.019)	1128	1.645 (0.020)	2874	2.401 (0.024)	1602	3.229 (0.043)
	3 months	5604	2.481 (0.019)	1128	1.685 (0.020)	2874	2.442 (0.023)	1602	3.112 (0.042)
	change	5604	-0.004 (0.010)	1128	0.041 (0.016)	2874	0.041 (0.014)	1602	-0.117 (0.024)
PD≥4	BL	5604	1094 (20%)	1128	6 (1%)	2874	467 (16%)	1602	621 (39%)
	3 months	5604	1052 (19%)	1128	11 (1%)	2874	466 (16%)	1602	575 (36%)
	change	5604	-42 (-1%)	1128	5 (0%)	2874	-1 (0%)	1602	-46 (-3%)
AL	BL	5602	2.100 (0.024)	1128	0.699 (0.024)	2872	1.899 (0.026)	1602	3.446 (0.049)
	3 months	5602	2.128 (0.024)	1128	0.747 (0.025)	2872	1.934 (0.026)	1602	3.448 (0.049)
	change	5602	0.028 (0.013)	1128	0.049 (0.018)	2872	0.034 (0.017)	1602	0.001 (0.031)
BOP	BL	5588	2834 (51%)	1128	241 (21%)	2858	1566 (55%)	1602	1027 (64%)
	3 months	5588	2830 (51%)	1128	214 (19%)	2858	1600 (56%)	1602	1016 (63%)
	change	5588	-4 (0%)	1128	-27 (-2%)	2858	34 (1%)	1602	-11 (-1%)
GI	BL	5604	3920 (70%)	1128	508 (45%)	2874	2130 (74%)	1602	1282 (80%)
	3 months	5604	3981 (71%)	1128	476 (42%)	2874	2158 (75%)	1602	1347 (84%)
	change	5604	61 (1%)	1128	-32 (-3%)	2874	28 (1%)	1602	65 (4%)
PI	BL	5597	3654 (65%)	1128	360 (32%)	2867	2155 (75%)	1602	1139 (71%)
	3 months	5597	3558 (64%)	1128	266 (24%)	2867	2030 (71%)	1602	1262 (79%)
	change	5597	-96 (-2%)	1128	-94 (-8%)	2867	-125 (-4%)	1602	123 (8%)

**Table 3:** Change from baseline to 3 months for apoptosis measurements.

	Time	All		Healthy		Mild CP		Mod/Sev CP	
		N Sites	Mean (SE) or N (%)	N Sites	Mean (SE) or N (%)	N Sites	Mean (SE) or N (%)	N Sites	Mean (SE) or N (%)
FasL	BL	659	222 (34%)	137	23 (17%)	331	128 (39%)	191	71 (37%)
	3 months	659	278 (42%)	137	20 (15%)	331	149 (45%)	191	109 (57%)
	change	659	56 (8%)	137	−3 (−2%)	331	21 (6%)	191	38 (20%)
Active FasL	BL	658	156 (24%)	137	27 (20%)	331	64 (19%)	190	65 (34%)
	3 months	658	151 (23%)	137	7 (5%)	331	73 (22%)	190	71 (37%)
	change	658	−5 (−1%)	137	−20 (−15%)	331	9 (3%)	190	6 (3%)
ProCas35K	BL	580	289 (50%)	98	34 (35%)	294	153 (52%)	188	102 (54%)
	3 months	580	180 (31%)	98	20 (20%)	294	86 (29%)	188	74 (39%)
	change	580	−109 (−19%)	98	−14 (−14%)	294	−67 (−23%)	188	−28 (−15%)
ProCas70K	BL	580	432 (74%)	98	65 (66%)	294	232 (79%)	188	135 (72%)
	3 months	580	441 (76%)	98	57 (58%)	294	238 (81%)	188	146 (78%)
	change	580	9 (2%)	98	−8 (−8%)	294	6 (2%)	188	11 (6%)
Active Cas	BL	580	220 (38%)	98	25 (26%)	294	116 (39%)	188	79 (42%)
	3 months	580	109 (19%)	98	2 (2%)	294	73 (25%)	188	34 (18%)
	change	580	−111 (−19%)	98	−23 (−23%)	294	−43 (−15%)	188	−45 (−24%)
ELISA	BL	426	0.696 (0.043)	10	0.182 (0.055)	237	0.643 (0.050)	179	0.795 (0.077)
	3 months	426	0.103 (0.015)	10	0.029 (0.009)	237	0.124 (0.024)	179	0.080 (0.015)
	change	426	−0.593 (0.044)	10	−0.153 (0.053)	237	−0.519 (0.051)	179	−0.715 (0.078)

**Table 4:** DNA Fragmentation Levels with Increasing PDs at Baseline.

Baseline PD	Baseline ELISA	
	Mean	SE
1	0.39	0.16
2	0.43	0.13
3	0.62	0.13
4	0.99	0.15
5	0.95	0.16
6+	1.45	0.21

Baseline: 1 < 4 (p=0.0011), 1 < 5 (p=0.0089), 1 < 6 (p<0.0001), 2 < 4 (p<0.0001), 2 < 5 (p=0.0008), 2 < 6 (p<0.0001), 3 < 4 (p=0.0214), 3 < 6 (p=0.0004).

**Table 5:** DNA Fragmentation Levels with Increasing PDs at 3 months.

3m PD	3m ELISA	
	Mean	SE
1	0.06	0.05
2	0.04	0.03
3	0.09	0.04
4	0.13	0.04
5	0.14	0.05
6	0.77	0.07

3m: 1–5 < 6 (p<0.0001).

**Table 6:** Apoptotic Protein Expression with increasing PDs at Baseline.

Baseline PD	Baseline									
	FasL		Active FasL		ProCas 35K		ProCas 70K		Active Cas	
	Mean	SE	Mean	SE	Mean	SE	Mean	SE	Mean	SE
1	35%	7%	23%	7%	33%	7%	68%	8%	24%	7%
2	30%	5%	22%	5%	46%	6%	72%	6%	36%	7%
3	32%	5%	21%	4%	49%	6%	77%	6%	34%	6%
4	36%	6%	24%	6%	55%	7%	81%	6%	48%	7%
5	49%	9%	35%	8%	59%	10%	82%	6%	49%	8%
6+	42%	9%	29%	8%	63%	10%	91%	5%	61%	9%

Baseline: No significant differences among groups for FasL (p=0.47), Active FasL (p=0.47), ProCas 35K (p=0.20), ProCas 70K (p=0.27), or Active Cas (p=0.11).

**Table 7:** Apoptotic Protein Expression with increasing PDs at 3 months.

3m PD	3m									
	FasL		Active FasL		ProCas 35K		ProCas 70K		Active Cas	
	Mean	SE	Mean	SE	Mean	SE	Mean	SE	Mean	SE
1	35%	7%	14%	4%	19%	6%	76%	7%	12%	4%
2	43%	6%	20%	4%	27%	6%	76%	6%	18%	5%
3	47%	6%	24%	5%	41%	7%	76%	6%	17%	4%
4	54%	7%	29%	7%	30%	6%	79%	5%	23%	5%
5	57%	10%	30%	6%	25%	7%	79%	8%	17%	8%
6+	60%	9%	50%	10%	51%	12%	70%	8%	34%	13%

3m: ProCas 35K 1 < 3 (p=0.0240). No significant differences among groups for FasL (p=0.23), Active FasL (p=0.13), ProCas 70K (p=0.71), or Active Cas (p=0.28).

**Table 8:** Correlations between pocket depth and DNA fragmentation/apoptosis.

		Baseline DNA fragmentation / apoptosis	with 3-month DNA fragmentation / apoptosis	3-month DNA fragmentation / apoptosis	with Change in DNA fragmentation / apoptosis	with Change in DNA fragmentation /apoptosis
PD	FasL	0.05	0.08	0.11	0.05	−0.04
	Active FasL	0.04	0.08	0.12	0.06	−0.03
	ProCas35K	0.08	0.04	0.04	−0.03	0.04
	ProCas70K	−0.02	0.01	0.02	0.02	0.00
	Active Cas	0.08	0.05	0.04	−0.03	0.05
	ELISA	0.05	−0.01	0.00	−0.05	−0.05
PD≥4	FasL	0.03	0.05	0.09	0.05	−0.01
	Active FasL	0.05	0.05	0.10	0.04	0.02
	ProCas35K	0.05	0.00	0.00	−0.04	0.04
	ProCas70K	−0.03	−0.01	0.01	0.03	−0.02
	Active Cas	0.06	0.01	0.00	−0.05	0.05
	ELISA	0.02	−0.02	0.00	−0.02	−0.01

**Table 9:** Analyses of sensitivity, specificity, positive predictive value (PPV) and negative predictive value (NPV).

		Sensitivity	Specificity	PPV	NPV
FasL	BL	38.1%	83.2%	89.6%	26.1%
	3months	49.4%	85.4%	92.8%	30.7%
Active FasL	BL	24.8%	80.3%	82.7%	21.9%
	3months	27.6%	94.9%	95.4%	25.6%
ProCas35K	BL	52.9%	65.3%	88.2%	22.0%
	3months	33.2%	79.6%	88.9%	19.5%
ProCas70K	BL	76.1%	33.7%	85.0%	22.3%
	3months	79.7%	41.8%	87.1%	29.5%
Active Cas	BL	40.5%	74.5%	88.6%	13.0%
	3months	22.2%	98.0%	98.2%	20.4%
